# Targeting Cholinergic Dysfunction and Gingipain Activity in Alzheimer’s Disease: A Dual AChE–Kgp Computational Study

**DOI:** 10.3390/ph19101589

**Published:** 2026-10-08

**Authors:** Ahmad M. Sait, Philip J. R. Day

**Affiliations:** 1 Medical Laboratory Science, Faculty of Applied Medical Science, King Abdulaziz University, Jeddah 21589, Saudi Arabia; ammsait@kau.edu.sa; 2 Regenerative Medicine Unit, King Fahd Medical Research Center, King Abdulaziz University, Jeddah 21589, Saudi Arabia; 3 Division of Evolution and Genomic Sciences, Faculty of Biology, Medicine and Health, The University of Manchester, Manchester M13 9PL, UK; 4 Department of Medicine, University of Cape Town, Cape Town 7925, South Africa

**Keywords:** Alzheimer’s disease (AD), *Porphyromonas gingivalis* (*P. gingivalis*), lysine-specific gingipain (Kgp)

## Abstract

**Introduction:** Alzheimer’s disease (AD) is a multifactorial neurodegenerative disorder for which single-target strategies may be insufficient. This study explored a host–microbial multitarget strategy using a CNS-oriented small molecule predicted to engage both human acetylcholinesterase (AChE) and *Porphyromonas gingivalis* lysine-specific gingipain K (Kgp). **Methods:** A CNS-focused library of 116,226 compounds was standardized and filtered to 1000 candidates for sequential AChE/Kgp docking. Refined docking, interaction-guided pose analysis, dual-target ranking, and SwissADME predictions were used for prioritization. ADKGP_0026671 was subsequently evaluated in single 100 ns molecular dynamics trajectories for AChE and Kgp, alongside donepezil and KYT-36 reference complexes, followed by MM/PBSA endpoint energy analysis. **Results:** ADKGP_0026671 showed docking scores of −11.804 kcal/mol for AChE and −7.345 kcal/mol for Kgp, was predicted to be BBB-permeant and not a P-glycoprotein substrate, and contained no PAINS or Brenk alerts. The single MD trajectories showed continued binding-site association and limited protein backbone deviation over the simulated period, although reproducibility cannot be established without independent replicas. MM/PBSA endpoint estimates were negative for AChE (−18.86 kcal/mol) and Kgp (−15.67 kcal/mol), but less favorable than donepezil and KYT-36, respectively. **Conclusions:** ADKGP_0026671 showed a balanced computational profile across two mechanistically distinct targets, supporting experimental evaluation of a host–microbial dual-target strategy in AD. These findings remain hypothesis-generating and require biochemical validation and replicated simulations.

## 1. Introduction

Alzheimer’s disease (AD) is a progressive neurodegenerative disorder considered to be one of the most common causes of cognitive decline. It has a multifactorial nature that is explained through the interaction of amyloid pathology, tau pathology, neuroinflammation, and synaptic dysfunction [1]. Anti-amyloid therapies for AD patients in the early stages attenuate cognitive and functional decline, but the benefits of treatment are modest and their risks are significant [1,2]. The multifactorial biology of AD consequently supports novel therapeutic concepts [3].

The cholinergic system also forms an important target in this disease. Acetylcholinesterase (AChE) terminates cholinergic neurotransmission by hydrolyzing acetylcholine, whilst degeneration of cholinergic neurons and reduced acetylcholine availability are seen in cognitive impairment in AD [4,5]. Inhibition of AChE reduces acetylcholine breakdown, thereby enhancing cholinergic neurotransmission. This mechanism underlies the therapeutic use of AChE inhibitors, including donepezil, galantamine, and rivastigmine, in AD [5,6]. Although their benefits are primarily symptomatic, AChE remains an established therapeutic target for the symptomatic management of AD [7].

A separate mechanistic route to develop AD is through chronic periodontal infection, specifically with *Porphyromonas gingivalis* (*P. gingivalis*). Periodontitis or infection with *P. gingivalis* has been linked to an increased risk of AD, potentially due to sustained inflammatory responses, promoting neuroinflammation and contributing to AD-related pathogenic changes [8,9]. *P. gingivalis* produces three major gingipains: lysine-specific gingipain K (Kgp), a cysteine protease, and the arginine-specific gingipains RgpA and RgpB [9,10]. These cysteine protease virulence factors contribute to host cell invasion, immune evasion, and tissue destruction. Kgp has been associated with AD-related pathology and has been investigated as a potential virulence-directed therapeutic target [9,11].

Several observations provide biological support for this association. *P. gingivalis* components, including its DNA, lipopolysaccharide, and gingipains, have been detected in brain tissue from patients with AD, with gingipains reported in regions associated with memory and in association with neurons, tau pathology, and intracellular amyloid-β; experimental oral infection has also produced neuroinflammation, amyloid accumulation, tau-related changes, and neurodegeneration in animal models [12].

Mechanistically, *P. gingivalis* may contribute to AD-related pathology through several converging processes, including disruption of blood–brain barrier integrity, chronic peripheral and central inflammation, microglial and astrocytic activation, oxidative stress, amyloid-β accumulation, and altered tau phosphorylation, although these findings remain largely associative or preclinical and do not yet establish a definitive causal role in human AD [13].

However, with multitarget strategies, researchers have increasingly investigated already known targets such as AChE and enzymes affecting neurotransmission or amyloid-related pathways [3,7]. Less investigated are *P. gingivalis* and Kgp, which have been implicated in a few neurodegeneration-relevant enzymatic processes and AD [8,9]. Currently, the two lines of research are carried out in widely separated ways. Investigation of a single compound capable of engaging both AChE and Kgp, which represent mechanistically distinct targets—a serine hydrolase and a lysine-specific cysteine protease, respectively—has not been widely explored. Combining cholinergic activity with targeting a *P. gingivalis-*mediated pathway could potentially improve the efficacy for the treatment of AD and related neurodegenerative diseases. An AD-oriented drug targeting *P. gingivalis* must feature CNS-compatible characteristics and preferably a high blood–brain barrier penetration [5].

In the current landscape, the idea of leveraging a virulence-targeted ligand of *P. gingivalis* in conjunction with cholinergic modulation is an attractive but unexplored therapeutic option. To avoid replacing conventional targets for AD entirely, as a proof of concept, we asked whether two targets with mechanistically distinct roles can be addressed using a single computationally prioritized scaffold. In a sequential computational approach combining a virtual screen and subsequent interaction-guided posing, ADME prioritization, molecular dynamics (MD) simulations, and MM/PBSA binding-energy analysis, we tried to locate one CNS-compatible small molecule with predicted dual-acting potential for human AChE and *P. gingivalis* Kgp.

## 2. Results

### 2.1. Docking Protocol Validation

Redocking of the co-crystallized AChE inhibitor donepezil reproduced the experimental binding geometry with a heavy-atom RMSD of 0.309 Å, supporting the ability of the AChE docking protocol to recover the crystallographic pose. In contrast, global redocking of KYT-36 into Kgp did not reproduce the crystallographic geometry, with a top-ranked pose RMSD of 9.695 Å and a best global RMSD of 6.211 Å. Local optimization initiated from the crystallographic KYT-36 pose yielded an RMSD of 0.976 Å, indicating that the prepared Kgp receptor could accommodate the experimentally observed binding geometry despite unsuccessful global pose reproduction. This limitation of Kgp global redocking was considered when interpreting subsequent docking predictions.

### 2.2. Dual-Target Virtual Screening and Final Candidate Prioritization

**Figure 1:**
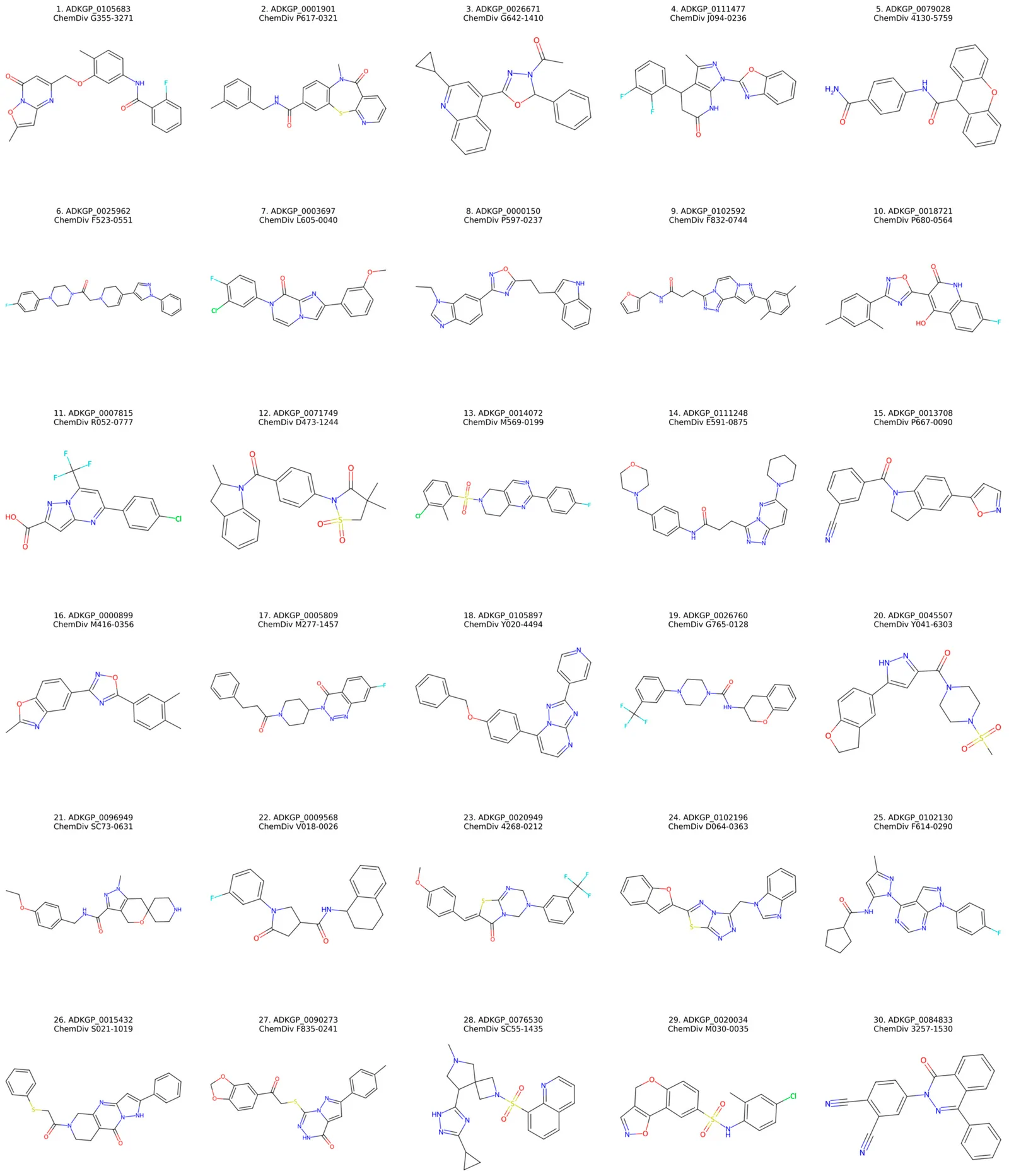
Two-dimensional chemical structures of the 30 dual-target candidates selected for refined docking against AChE and Kgp. Compounds are presented according to their final dual-target consensus rank and labeled with the corresponding project identifier and ChemDiv catalog number. Atom colors follow standard chemical drawing conventions: carbon (black), oxygen (red), nitrogen (blue), sulfur (yellow), and halogens (light green/cyan).

The CNS-focused chemical collection comprised 116,226 chemical records. Following standardization, physicochemical and medicinal-chemistry filtering, CNS-focused prioritization, structural clustering, and representative compound selection, 1000 compounds were retained for virtual screening. Sequential docking against AChE and Kgp, followed by dual-target consensus ranking, identified 30 compounds for refined docking against both targets (Supplementary Table S1). Chemical identifiers for these 30 candidates, including their ChemDiv catalog numbers, canonical SMILES, and InChIKeys, are provided in Supplementary Table S2, with their corresponding 2D chemical structures shown in (Figure 1). Refined docking of the 30 compounds against both AChE and Kgp generated 296 poses across 60 target–ligand combinations. All poses were evaluated using PLIP-assisted interaction analysis and the predefined preferred-pose selection criteria. One preferred pose was selected for each of the 60 target–ligand combinations: Mode 1 was retained for 34 combinations, whereas an alternative pose was selected for the remaining 26 (Supplementary Table S3).

### 2.3. Predicted Binding Modes of ADKGP_0026671 in AChE and Kgp

Although ADKGP_0026671 ranked third in the docking-based dual-target consensus, final candidate selection was based on a secondary CNS-oriented decision framework rather than consensus rank alone. Among the five highest-ranked compounds, ADKGP_0026671 was the only candidate predicted to cross the blood–brain barrier while also being predicted not to be a P-glycoprotein substrate. It also showed the most favorable preferred Kgp docking score among the five finalists (−7.345 kcal/mol), while retaining competitive AChE binding (−11.804 kcal/mol) and a predicted Kgp catalytic-site contact. Its CNS-oriented physicochemical profile included a TPSA of 54.79 Å^2^, with no PAINS or Brenk alerts. Although ADKGP_0026671 showed one Lipinski-rule violation due to MLOGP > 4.15, this single deviation did not preclude CNS-oriented follow-up because the compound retained favorable predicted BBB permeability, was not predicted to be a P-glycoprotein substrate, and otherwise showed a compatible physicochemical profile. In contrast, the first- and second-ranked compounds were not predicted to cross the blood–brain barrier, while the fourth-ranked compound was predicted to be a P-glycoprotein substrate. ADKGP_0026671 was therefore selected for molecular dynamics analysis because it provided the most favorable overall combination of dual-target docking compatibility, Kgp engagement, and CNS-oriented ADME characteristics among the top five candidates (Table 1).

### 2.4. Binding Mode of ADKGP_0026671

**Figure 2:**
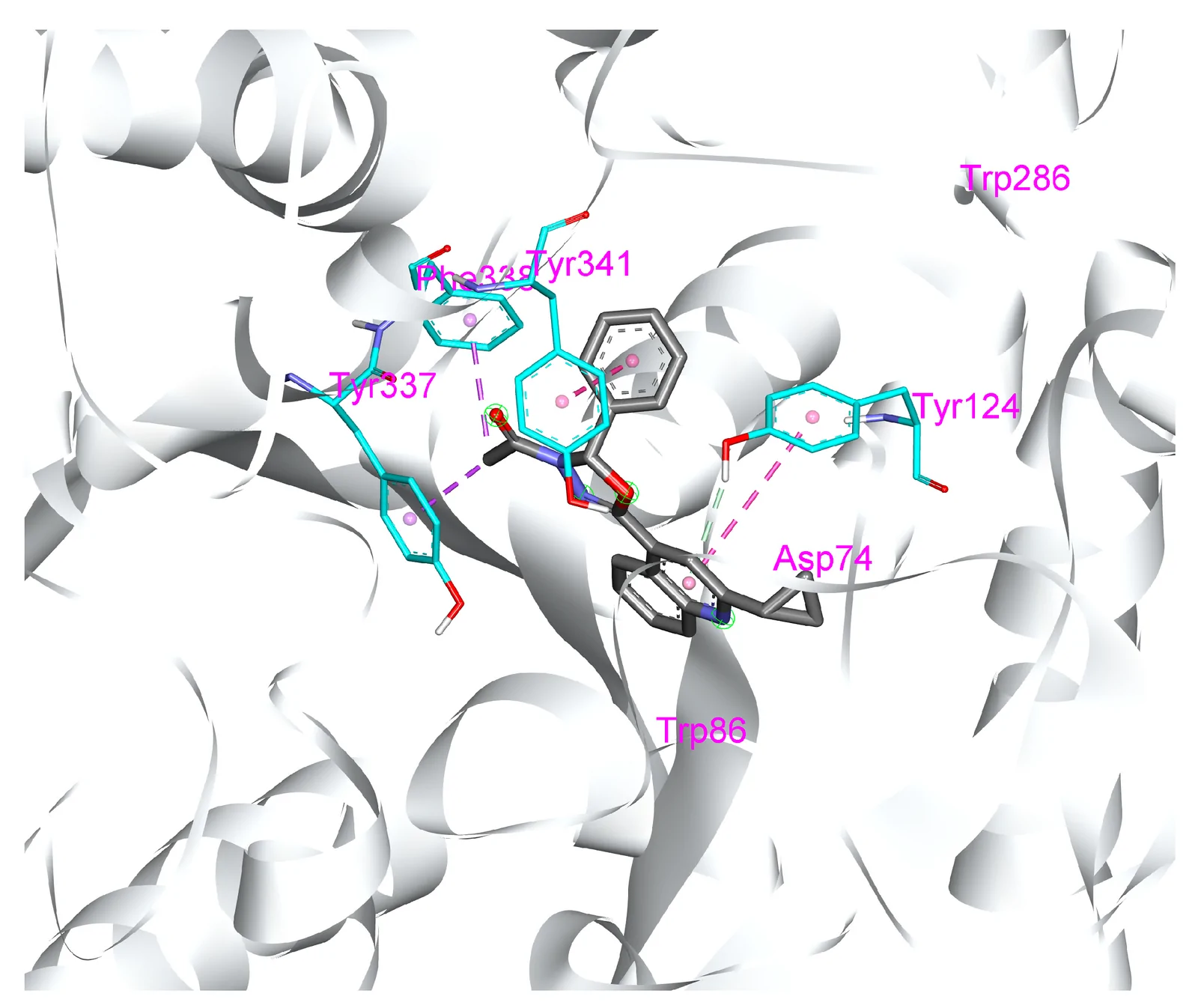
Predicted binding mode of ADKGP_0026671 within acetylcholinesterase (AChE). The docked ligand is positioned within the AChE binding gorge and surrounded by key residues, including Tyr124, Tyr337, Phe338, and Tyr341. The protein is represented as a grey cartoon, with selected interacting residues labelled in magenta. The ligand and selected residues are shown as sticks, with heteroatoms coloured according to standard chemical conventions. Circles and dashed lines represent predicted molecular interactions.

**Figure 3:**
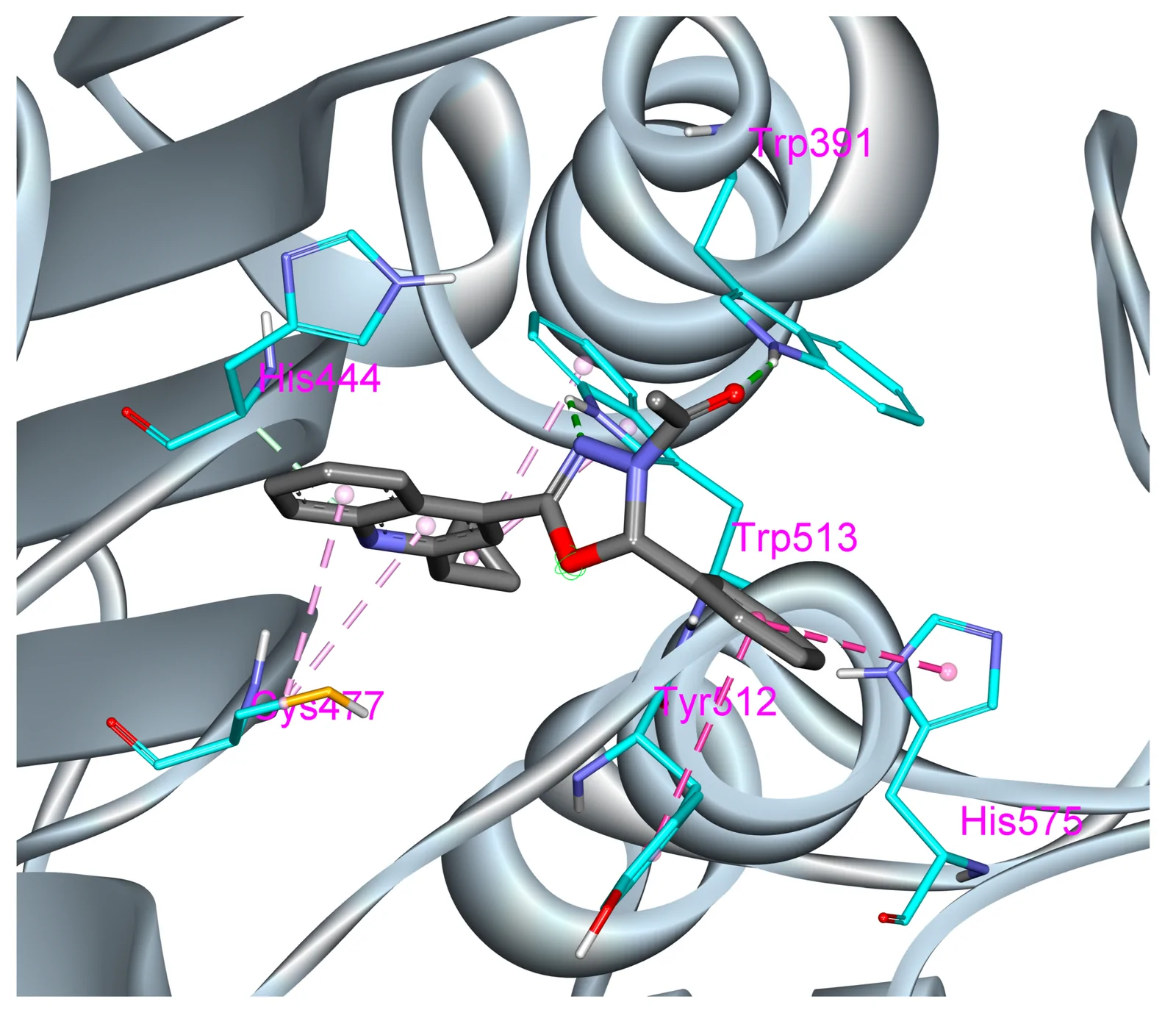
Predicted binding mode of ADKGP_0026671 within gingipain K (Kgp). The ligand occupies the Kgp catalytic cleft and interacts with Trp391, His444, Cys477, Trp513, Tyr512, and His575. PLIP identified a backbone hydrogen bond with Cys477 (3.98 Å), without involvement of the catalytic thiol side chain. The protein is represented as a grey cartoon, while the ligand and selected binding-site residues are displayed as sticks. Residue labels are shown in magenta, and atoms are coloured according to standard chemical conventions. Magenta dashed lines and circular markers indicate predicted molecular interactions.

ADKGP_0026671 could be accommodated within the binding pockets of AChE and Kgp upon molecular docking analysis (Figure 2 and Figure 3). In AChE, it occupied the active-site gorge and formed predicted contacts with aromatic residues including Tyr124, Tyr337, Phe338, and Tyr341. The selected pose also showed direct contacts with the catalytic-triad residues Ser203 and His447, whereas no direct interaction with Glu334 was identified. Thus, the predicted binding mode involved both the catalytic region and surrounding aromatic gorge residues, rather than only peripheral pocket stabilization. Additional non-covalent contacts with neighboring residues further supported stabilization of the ligand within the AChE binding pocket. The preferred AChE pose engaged residues associated with both the CAS and PAS regions, as detailed in Supplementary Table S3. Because docking alone cannot establish inhibition kinetics, the competitive, non-competitive, or mixed mechanism of ADKGP_0026671 remains to be determined experimentally.

Similarly, the preferred docked pose positioned ADKGP_0026671 within the Kgp catalytic binding cleft and generated predicted contacts with residues including Trp391, His444, Cys477, Trp513, Tyr512, and His575 (Figure 3). Residue-level PLIP analysis identified a predicted hydrogen bond involving Cys477, with a donor–acceptor distance of 3.98 Å and a donor angle of 143.61°. This interaction was backbone-mediated rather than involving the Cys477 side-chain thiol. Additional hydrophobic/aromatic and polar contacts with surrounding residues contributed to stabilization of the predicted complex. Thus, the docking pose supports direct contact with Cys477 within the catalytic region but does not demonstrate covalent engagement or catalytic inhibition.

### 2.5. Protein Structural Stability During Molecular Dynamics

**Figure 4:**
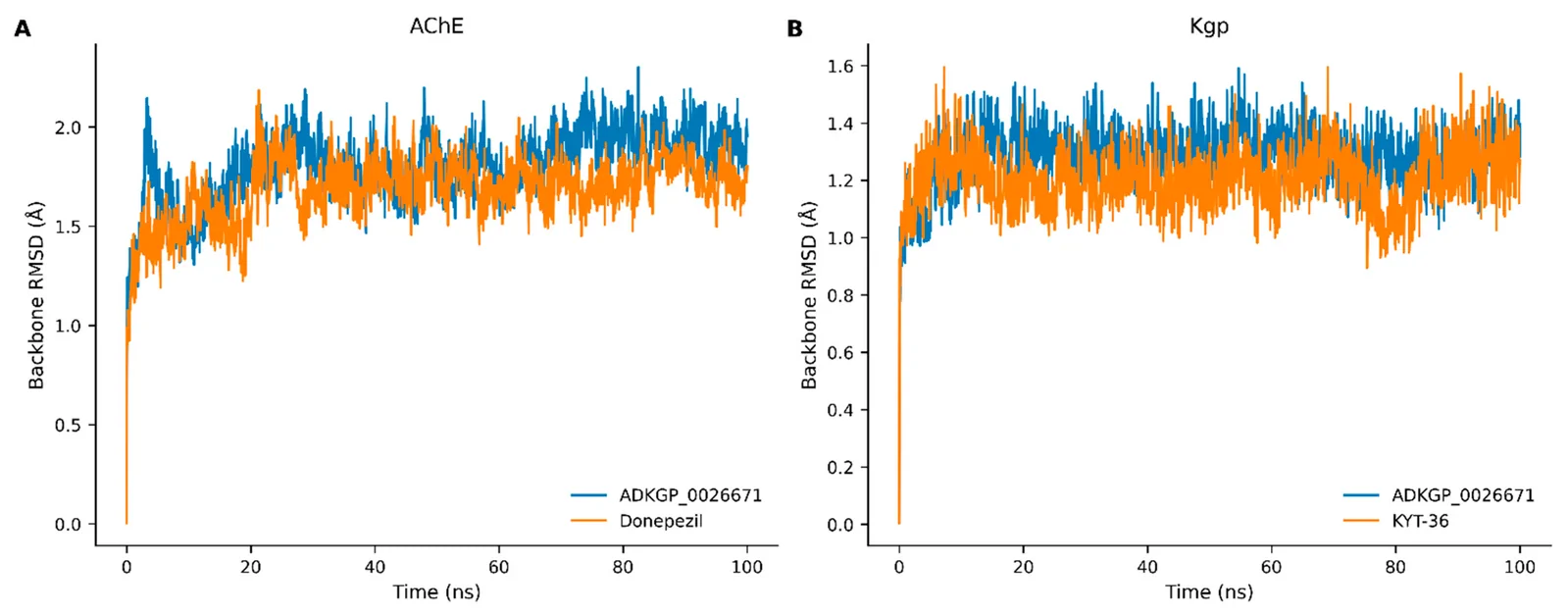
Backbone RMSD profiles of AChE and Kgp complexes during 100 ns MD simulations. (**A**) AChE–ADKGP_0026671 and AChE–donepezil. (**B**) Kgp–ADKGP_0026671 and Kgp–KYT-36.

During the early phase of the AChE simulations, both the ADKGP_0026671 and donepezil complexes showed an initial increase in backbone RMSD, followed by relatively consistent backbone behavior (Figure 4A). Quantitative analysis over the 20–100 ns interval gave mean backbone RMSD values of 1.86 ± 0.13 Å for AChE–ADKGP_0026671 and 1.74 ± 0.11 Å for AChE–donepezil. The ADKGP_0026671 complex therefore showed a slightly higher average backbone deviation than the donepezil reference, but no progressive structural drift was observed. For Kgp, mean backbone RMSD values over the same interval were 1.29 ± 0.08 Å for Kgp–ADKGP_0026671 and 1.20 ± 0.10 Å for Kgp–KYT-36 (Figure 4B). These results indicate limited backbone deviation in all four trajectories during the analyzed interval.

**Figure 5:**
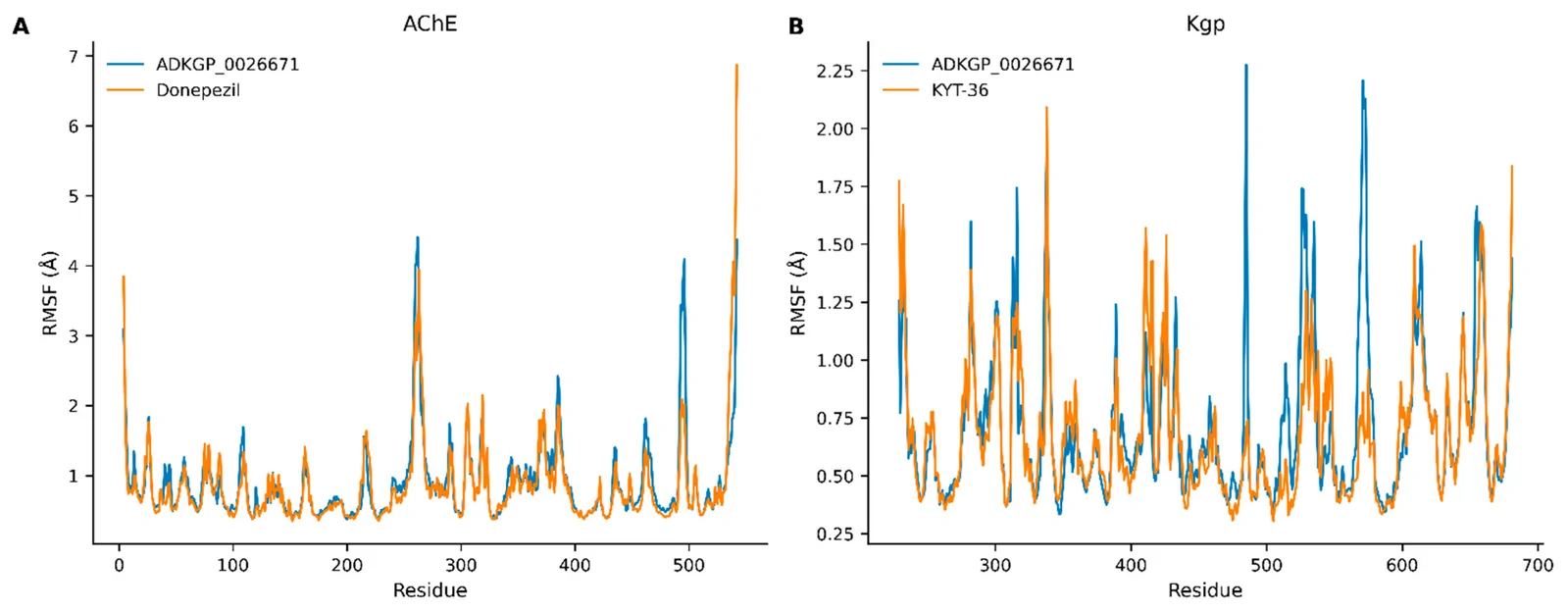
Cα RMSF profiles of AChE and Kgp complexes during 100 ns MD simulations. (**A**) AChE–ADKGP_0026671 and AChE–donepezil. (**B**) Kgp–ADKGP_0026671 and Kgp–KYT-36.

Cα RMSF values were also quantified over the 20–100 ns interval. For AChE, mean residue-level RMSF values were 0.80 ± 0.54 Å for the ADKGP_0026671 complex and 0.81 ± 0.60 Å for the donepezil complex. The RMSF profiles were closely comparable across most of the protein, with larger fluctuations confined to discrete flexible regions (Figure 5A), indicating broadly similar residue-level flexibility between the candidate and reference complexes.

A similar pattern was observed for Kgp, with mean residue-level Cα RMSF values of 0.67 ± 0.32 Å for Kgp–ADKGP_0026671 and 0.65 ± 0.27 Å for Kgp–KYT-36. The RMSF profiles largely overlapped across most residues, with only localized differences at individual regions (Figure 5B). Overall, the quantitative RMSD and RMSF analyses indicate broadly comparable protein structural behavior between ADKGP_0026671 and the corresponding reference inhibitor complexes during the analyzed 20–100 ns interval. As these observations are derived from a single 100 ns trajectory for each complex, comparisons of structural stability should be regarded as preliminary and descriptive of the individual trajectories rather than evidence of reproducible differences between systems.

### 2.6. Residue-Level Flexibility During Molecular Dynamics

The RMSF of AChE in the ADKGP_0026671 and donepezil complexes had close profiles along most of the protein. The low fluctuations involved most of the residues, and few peaks could be seen in both binding situations. The largest fluctuations happened at flexible areas in discrete sites, and both profiles were reasonably close (Figure 5A). The profile in both situations showed large fluctuations on some amino residues, with no significant differences seen generally except in the area where most residues are located. So, it showed that ADKGP_0026671 would not cause the AChE flexibility to change from donepezil in a general way.

Similarly, Kgp also showed the same pattern and RMSF profile of the complexes of ADKGP_0026671 and KYT-36, largely superimposed on each other for most of the residues (Figure 5B). At several positions, local differences in the RMSF could be observed, such as during the conformation, a region showed transiently higher fluctuation in the ADKGP_0026671 complex, but again restricted to a segment and not pertaining to the whole length of the protein. Overall, RMSF shows ADKGP_0026671 to have residue-level dynamics that are largely similar compared to respective reference inhibitors.

### 2.7. Ligand Stability and Binding-Pocket Retention

**Figure 6:**
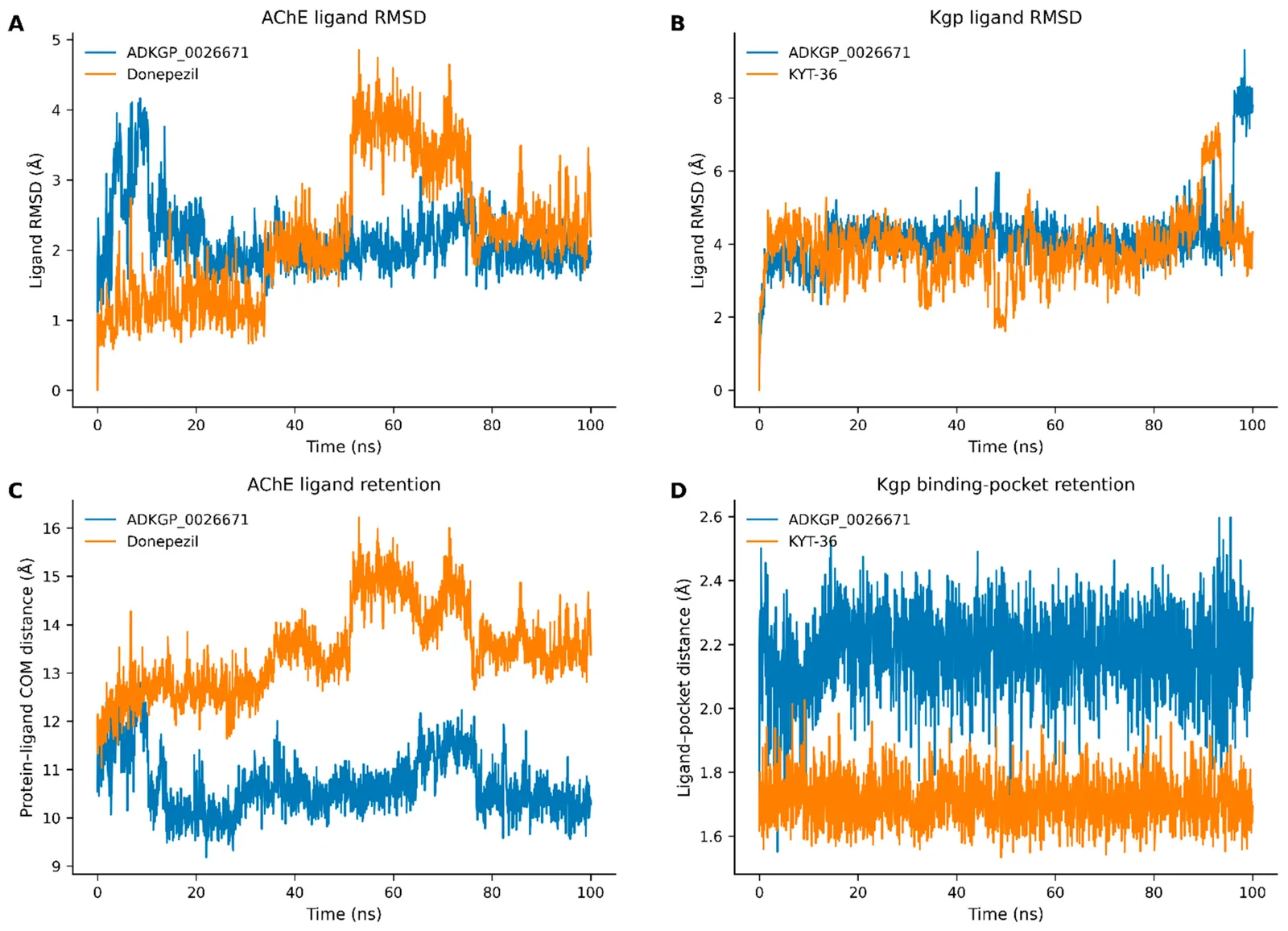
Ligand conformational stability and binding-pocket retention in 100 ns molecular dynamics simulations. Ligand RMSD profiles of (**A**) AChE-ADKGP_0026671 and AChE-donepezil and (**B**) Kgp-ADKGP_0026671 and Kgp-KYT-36 are presented. Binding-region retention was evaluated by (**C**) protein–ligand center-of-mass (COM) distance for AChE complexes and (**D**) binding pocket distance for Kgp complexes, combined with the ligand RMSD, to differentiate ligand reorientation within the binding region from possible displacement outside.

During the AChE–ADKGP_0026671 simulation, the ligand underwent an initial conformational adjustment within the first 10–15 ns, after which its ligand RMSD remained largely within ~1.8–2.5 Å for the remainder of the trajectory (Figure 6A). The protein–ligand center-of-mass (COM) distance also remained relatively stable, generally within ~10–11.5 Å, indicating continued association with the AChE binding-site region throughout the simulated period. In the AChE–donepezil complex, a larger conformational change was observed at approximately 50–75 ns, during which ligand RMSD increased together with the protein–ligand COM distance before both partially returned toward their earlier ranges (Figure 6C).

For Kgp, both ADKGP_0026671 and KYT-36 showed ligand RMSD values generally within the 3–5 Å range over most of the trajectory (Figure 6B). Toward the end of the simulation, ADKGP_0026671 exhibited a marked increase in ligand RMSD, approaching 8 Å, without a corresponding increase in the ligand–pocket distance, which remained broadly within ~2.0–2.3 Å throughout the trajectory (Figure 6D). Because ligand RMSD alone cannot distinguish intrapocket rearrangement from partial disengagement, this late-stage behavior was further examined using time-resolved contact-occupancy and pocket-proximity analyses (Figure S1). Several predefined Kgp binding-site contacts were retained during the 80–100 ns interval, including high occupancy of Trp391, Tyr512, and Trp513, together with continued contacts involving Cys477 and His444. In addition, the minimum ligand–pocket distance remained below the 4.0 Å contact threshold throughout the late trajectory (Figure S2). These findings indicate that ADKGP_0026671 remained associated with the Kgp binding region despite a substantial change from its initial pose. The late RMSD increase is therefore interpreted as a major conformational rearrangement within or near the binding region rather than evidence of complete dissociation during the simulated 100 ns; however, partial disengagement or subsequent dissociation on longer timescales cannot be excluded. KYT-36 maintained a comparatively lower ligand–pocket distance of approximately 1.6–1.8 Å. Hydrogen-bond persistence analysis over the 20–100 ns interval showed that ADKGP_0026671 did not maintain a conventional persistent hydrogen bond with AChE under the applied geometric criteria. Protein–ligand heteroatom distances of ≤3.5 Å were observed in 8.6% of analyzed frames, while distances of ≤4.0 Å occurred in 57.0% of frames, indicating intermittent polar proximity rather than a sustained hydrogen-bond interaction.

### 2.8. MM/PBSA Binding-Energy Analysis

For all four complexes, the MM/PBSA analyses generated favorable, negative binding-energy estimates (Table 2). For AChE, ADKGP_0026671 showed a mean ΔTOTAL of −18.86 kcal/mol, compared with −30.65 kcal/mol for donepezil. Van der Waals interactions contributed favorably in both systems, whereas polar solvation opposed binding. Donepezil showed a markedly stronger favorable electrostatic contribution, which was partly offset by a correspondingly unfavorable polar solvation term.

For Kgp, ADKGP_0026671 showed a mean ΔTOTAL of −15.67 kcal/mol, whereas KYT-36 showed −42.74 kcal/mol. Again, van der Waals interactions were favorable for both systems, while the strong electrostatic contribution of KYT-36 was substantially offset by polar solvation. The ΔTOTAL standard deviation was larger for Kgp–KYT-36 (15.22 kcal/mol) than for Kgp–ADKGP_0026671 (3.56 kcal/mol), indicating greater within-trajectory energetic variability across the analyzed snapshots.

After accounting for temporal autocorrelation, the corrected SEM values were 0.79 kcal/mol for AChE–ADKGP_0026671, 0.75 kcal/mol for AChE–donepezil, 0.47 kcal/mol for Kgp–ADKGP_0026671, and 2.91 kcal/mol for Kgp–KYT-36. Overall, ADKGP_0026671 retained favorable endpoint binding-energy estimates for both targets, although both reference inhibitors showed more negative mean ΔTOTAL values. These results therefore support energetic compatibility with both binding sites rather than higher binding affinity than the reference inhibitors.

## 3. Discussion

AD is a complex, multifactorial disorder that evades therapeutic intervention. Targeting one pathway has proven insufficient, but there is now growing interest in multi-target-directed ligands (MTDLs), which have a combination of complementary mechanisms [14]. AChE inhibition with other AD-relevant targets has been well established as MTDL-based strategies. Acetylcholinesterase/beta-site amyloid precursor protein cleaving enzyme 1 (AChE/BACE-1) dual inhibition has been reported, with acetylcholinesterase/monoamine oxidase B(AChE/MAO-B) inhibition that has also been reported, aiming to treat the cholinergic and monoaminergic and oxidative stress-associated pathways [15,16]. Another design inhibits AChE and kinases like GSK-3β, which phosphorylates tau [17].

When designing MTDLs, a balance of affinities towards the complementary targets is ideal instead of maximal potency toward a singular target. The AChE–Kgp model described in this work extends the concept further towards that of a ‘host–microbe’ pair; instead of two human targets, one drug interacts with the symptomatic target of AChE and Kgp, which is a protease secreted by *P. gingivalis*. This hypothesis is supported by Dominy et al. [18], who observed gingipains in AD brain specimens in association with tau and ubiquitin pathology, while in mouse models, small-molecule inhibitors of gingipains attenuated the brain *P. gingivalis* load, inhibited the formation of Aβ42, reduced neuroinflammation, and rescued neurons. This is a novel MTDL in that it pairs an established cholinergic target with a microorganism proposed to contribute toward AD development.

We compared our candidate ADKGP_0026671 directly with benchmark inhibitors optimized for each target. As expected, donepezil (an AChE-specific inhibitor) showed stronger computed binding at AChE than ADKGP_0026671 (MM/PBSA ≈ −30.7 vs. −18.9 kcal/mol). Within the individual 100 ns trajectories examined, both AChE complexes displayed apparent structural stability; ADKGP_0026671 remained within the AChE active site throughout the simulation, while donepezil exhibited a modest ligand rearrangement midway through the trajectory. These observations are preliminary because independent MD replicates were not performed. Likewise, at Kgp, the reference inhibitor KYT-36 (a potent Kgp inhibitor with Ki ≈ 10^−10^ M) yielded a more favorable binding energy (−42.7 kcal/mol vs. −15.7 for ADKGP_0026671), while ADKGP_0026671 remained within the Kgp pocket during the simulated trajectory. Its late-stage increase in ligand RMSD was accompanied by continued binding-site contacts and a stable ligand–pocket distance, indicating that the ligand remained associated with the Kgp binding region during the simulated trajectory; however, partial disengagement or subsequent dissociation on longer timescales cannot be excluded. Within these single trajectories, the MM/PBSA binding energies for ADKGP_0026671 showed lower variability than those of KYT-36 (SD 3.6 vs. 15.2 kcal/mol); however, this difference should not be interpreted as evidence of greater reproducible stability without replicate simulations. The target-specific inhibitors (donepezil and KYT-36) showed more favorable, calculated binding energies at their respective targets, whereas ADKGP_0026671 maintained predicted contacts with both proteins during the trajectories examined. This balance of predicted binding potential across targets is consistent with the objective of MTDL design.

Clinical trial precedence illustrates that gingipain inhibition has yet to provide a robust response. Atuzaginstat/COR388 failed to generate clinically significant improvements in the overall intent-to-treat population in the 643-participant GAIN trial (Phase 2/3); however, reduced cognitive decline was observed in prespecified *P. gingivalis infection* +ve subgroups, suggesting gingipain targeting therapy has not been verified in AD yet [18]. The generated results can enable experimental study of how host innate immunity works against this pathogenic protein from *P. gingivalis* (ADKGP_0026671), exploring the feasibility of the dual-target hypothesis of the host–microbe model.

## 4. Materials and Methods

### 4.1. Target Structures and Receptor Preparation

The X-ray crystal structures of human acetylcholinesterase (AChE) in complex with donepezil (PDB ID 4EY7; resolution 2.35 Å) and Porphyromonas gingivalis lysine-specific gingipain K (Kgp) in complex with KYT-36 (PDB ID 6I9A; resolution 1.20 Å) were selected as the receptor structures [19,20]. 4EY7 was chosen because it represents human AChE in a ligand-bound conformation with the clinically established inhibitor donepezil occupying the active-site gorge, thereby providing an experimentally defined binding geometry suitable for docking-site definition and protocol validation. 6I9A was selected because it provides a high-resolution inhibitor-bound structure of Kgp, with KYT-36 occupying the catalytic binding region, enabling precise definition of the Kgp docking site and comparison with a structurally characterized reference inhibitor. Chain B was retained as the representative protein chain for both structures. Before receptor preparation, the selected chains were inspected for missing atoms and residues, alternate conformations, chain discontinuities, and preservation of the catalytic and ligand-binding regions.

Proteins were protonated at pH 7.4 using PDB2PQR with PROPKA-based pKa assignment [21,22] and converted to PDBQT using Meeko. Docking boxes were centered at (10.945, −56.551, −23.468 Å; 20 × 28 × 18 Å) for AChE and (−9.104, −61.820, 42.277 Å; 26 × 24 × 22 Å) for Kgp, encompassing their respective catalytic and ligand-binding regions.

Docking-protocol validation was performed by redocking the co-crystallized reference ligands into their respective binding sites. Donepezil was redocked into AChE (PDB 4EY7), and KYT-36 was redocked into Kgp (PDB 6I9A) using the same receptor preparation and docking-box definitions employed for virtual screening. Heavy-atom RMSD between the predicted and crystallographic ligand poses was calculated after structural alignment. In addition, because global redocking of KYT-36 did not reproduce the crystallographic pose, a local optimization starting from the experimental KYT-36 geometry was performed to assess compatibility of the prepared Kgp receptor with the crystallographic binding mode.

### 4.2. Chemical Library and Virtual Screening

The screening collection combined four commercially curated ChemDiv CNS-focused collections: the CNS Library, CNS BBB Library, CNS gMPO Library, and CNS Targets Focused Library, yielding 116,226 records. The four collections represent complementary CNS-oriented chemical spaces, including general CNS-focused chemistry, BBB-oriented compounds, multiparameter-optimized CNS-like compounds, and compounds prioritized toward CNS protein targets. No separate version identifiers were provided with the source files used in this study. Structures were standardized to parent single-fragment forms, with disconnected salts and counterions removed. Stereochemical information present in the source structures was retained, and no additional stereochemical enumeration was performed.

Structures were then filtered for physicochemical properties, drug-likeness, and structural alerts, with compounds triggering PAINS or Brenk alerts excluded. CNS-oriented prioritization was performed using a project-specific four-component CNS preference score (CNS_PREF4) based on cLogP, molecular weight (MW), topological polar surface area (TPSA), and hydrogen-bond donors (HBD). The cLogP component received a maximum score of 1.0 for cLogP ≤ 3.0 and decreased linearly to zero between 3.0 and 5.0. The MW component received a maximum score of 1.0 for MW ≤ 360 Da and decreased linearly to zero between 360 and 500 Da. The TPSA component received a maximum score of 1.0 between 40 and 90 Å^2^, with linear penalties below 40 Å^2^ and above 90 Å^2^, reaching zero at 20 and 120 Å^2^, respectively. The HBD component was scored as 1.00, 0.833, 0.500, and 0.167 for 0, 1, 2, and 3 HBDs, respectively. The four component scores were summed to give CNS_PREF4, and compounds with CNS_PREF4 ≥ 3 were prioritized. Diversity selection was then applied to produce 1000 non-duplicate compounds. The selected compounds were prepared at pH 7.4 using MolScrub v0.2.2, followed by RDKit ETKDGv3 three-dimensional conformer generation and MMFF94s optimization. The final structures used for docking were represented as PDBQT files generated with Meeko using Gasteiger partial charges.

The virtual-screening workflow was implemented as a hierarchical enrichment strategy rather than as a symmetric two-target screen. AChE was used as the primary screening target because it represents the established cholinergic target in AD, while Kgp was incorporated as a second, mechanistically distinct target to identify compounds with additional predicted compatibility toward a *P. gingivalis* virulence factor. Accordingly, the 1000 CNS-focused compounds were first docked against AChE using AutoDock Vina 1.2.7 at an exhaustiveness of 8 [23]. The top 100 AChE-ranked compounds were then advanced to Kgp screening under the same docking conditions. This sequential filtering strategy may therefore have excluded genuine dual-target binders that showed comparatively weaker predicted binding to AChE and consequently did not progress to Kgp screening. Within this AChE-enriched subset, target-specific ranks were normalized and integrated to generate a dual-target consensus ranking. The top 30 compounds were subsequently subjected to refined docking against both AChE and Kgp at an exhaustiveness of 32, with up to five poses retained per ligand and a fixed random seed.

### 4.3. Interaction Guided Dual-Target Prioritization

Pose refinement included analysis of hydrogen bonds, hydrophobic contacts, π-stacking, π-cation interactions, salt bridges, and halogen bonds, as well as other PLIP-supported interactions through the protein–ligand interaction profiler [24]. Functional site annotations for human acetylcholinesterase (hAChE) and Kgp were: Ser203, Glu334, His447, Trp86, Trp286, Tyr337, Phe338, Tyr341, Tyr72, Asp74, Tyr124 and Asp388, His444, Cys477, Trp391, Gly445, Ala451, Tyr512, Trp513, Asp516, His575, respectively. Mode 1 was retained as the docking-score reference for quantitative compound ranking. Alternative poses were considered only for interaction-based structural interpretation and only when their Vina score was within 0.5 kcal/mol of Mode 1. Among these near-equivalent poses, an alternative pose could be selected when it showed improved engagement of the predefined functionally relevant binding-site residues. Selection favored greater functional-site category coverage or at least two additional predefined important-residue contacts; where these criteria were otherwise equivalent, the more favorable Vina score and then the lower mode number were used as tie-breakers. Total interaction count alone was not used to override the docking-score criterion.

Importantly, preferred-pose selection was not used to calculate the quantitative dual-target ranking. For each target, compounds were ranked independently using their refined Mode-1 Vina scores. Target-specific ranks were normalized as (*r −* 1)/(*N* − 1)(*r* − 1)/(*N* − 1), where *r* is the target-specific rank and *N* = 30 *N* = 30, and the AChE and Kgp normalized ranks were averaged to generate the dual-target consensus score. The PLIP-guided preferred poses were subsequently used to characterize plausible binding orientations and functionally relevant protein–ligand interactions.

### 4.4. ADME and CNS-Oriented Selection

To ensure quality, the physicochemical, PAINS/Brenk, and CNS-related properties of the final thirty compounds were re-evaluated. The top five candidates underwent further examination with SwissADME. This focused on their predicted gastrointestinal absorption, their ability to predict blood–brain barrier penetration, whether they acted as predicted P-glycoprotein substrates, and their overall drug and potential likeness alongside medicinal-chemistry alerts [25]. The final selection process incorporated evidence from dual-target docking and PLIP alongside these CNS-relevant ADME forecasts. ADKGP_0026671 emerged as the chosen compound due to its advantageous overall characteristics. These included predicted blood–brain barrier permeation, confirmation that it is not a P-glycoprotein substrate, a molecular weight of 357.41, a TPSA of 54.79 Å^2,^ and, crucially, no PAINS or Brenk alerts.

### 4.5. Molecular Dynamics Simulations and MM/PBSA Analysis

Four molecular dynamics simulations were performed: AChE–ADKGP_0026671, AChE–donepezil, Kgp–ADKGP_0026671, and Kgp–KYT-36. The selected docked pose of ADKGP_0026671 was used, whereas crystallographic poses were retained for the reference inhibitors. Proteins were protonated at pH 7.4 using PDB2PQR/PROPKA [21,22], and parameterized with Amber99SB-ILDN [26]. For Kgp, Asp388 was modeled in its deprotonated carboxylate form, His444 as a neutral histidine protonated at Nε2 (HIE), and Cys477 in its neutral thiol form. Ligands were parameterized using GAFF2 with AM1-BCC partial charges assigned using Antechamber/ACPYPE [27,28]. Each complex was centered in a rhombic dodecahedral periodic box with a minimum solute-to-box-edge distance of 1.0 nm, solvated with TIP3P water, neutralized, and adjusted to approximately 0.15 M NaCl. Periodic boundary conditions were applied in all three spatial dimensions. Long-range electrostatic interactions were treated using particle-mesh Ewald electrostatics with a 1.0 nm real-space Coulomb cutoff, 0.12 nm Fourier spacing, fourth-order interpolation, and an Ewald relative tolerance of 1 × 10^−5^. Van der Waals interactions used a 1.0 nm cutoff with potential-shift treatment. Bond constraints were applied using LINCS (order 4; one iteration), with a 2 fs integration timestep. Following energy minimization, systems underwent 100 ps NVT equilibration at 300 K using the V-rescale thermostat, followed by at least 500 ps NPT equilibration at 300 K and 1 bar using isotropic C-rescale pressure coupling. Kgp–KYT-36 received an additional 500 ps NPT equilibration extension. Unrestrained 100 ns production simulations were subsequently performed. Compressed coordinates were written every 10 ps, and trajectories used for downstream structural analyses were processed at 50 ps intervals. All EM, NVT, NPT, and production simulations were performed with GROMACS 2025.3 on the Aziz Supercomputer at King Abdulaziz University, Jeddah.

MM/PBSA calculations were performed using gmx_MMPBSA v1.6.5 on 201 snapshots extracted at 250-ps intervals from the final 50 ns of each trajectory using the single-trajectory approach [29]. The calculated energetic components included van der Waals, electrostatic, polar solvation, and nonpolar solvation terms. Poisson–Boltzmann calculations used an internal dielectric constant of 1.0, an external solvent dielectric constant of 80.0, an ionic strength of 0.150 M, and a fill ratio of 4.0. Atomic radii were taken from the AMBER topology (radiopt = 0), with the mbondi2 radii set assigned during topology conversion. The default PB nonpolar solvation treatment was used (inp = 2). No per-residue energy decomposition was performed. Configurational entropy was not evaluated; consequently, the reported ΔTOTAL values were interpreted as MM/PBSA endpoint binding-energy estimates rather than rigorous absolute binding free energies.

**Figure 7:**
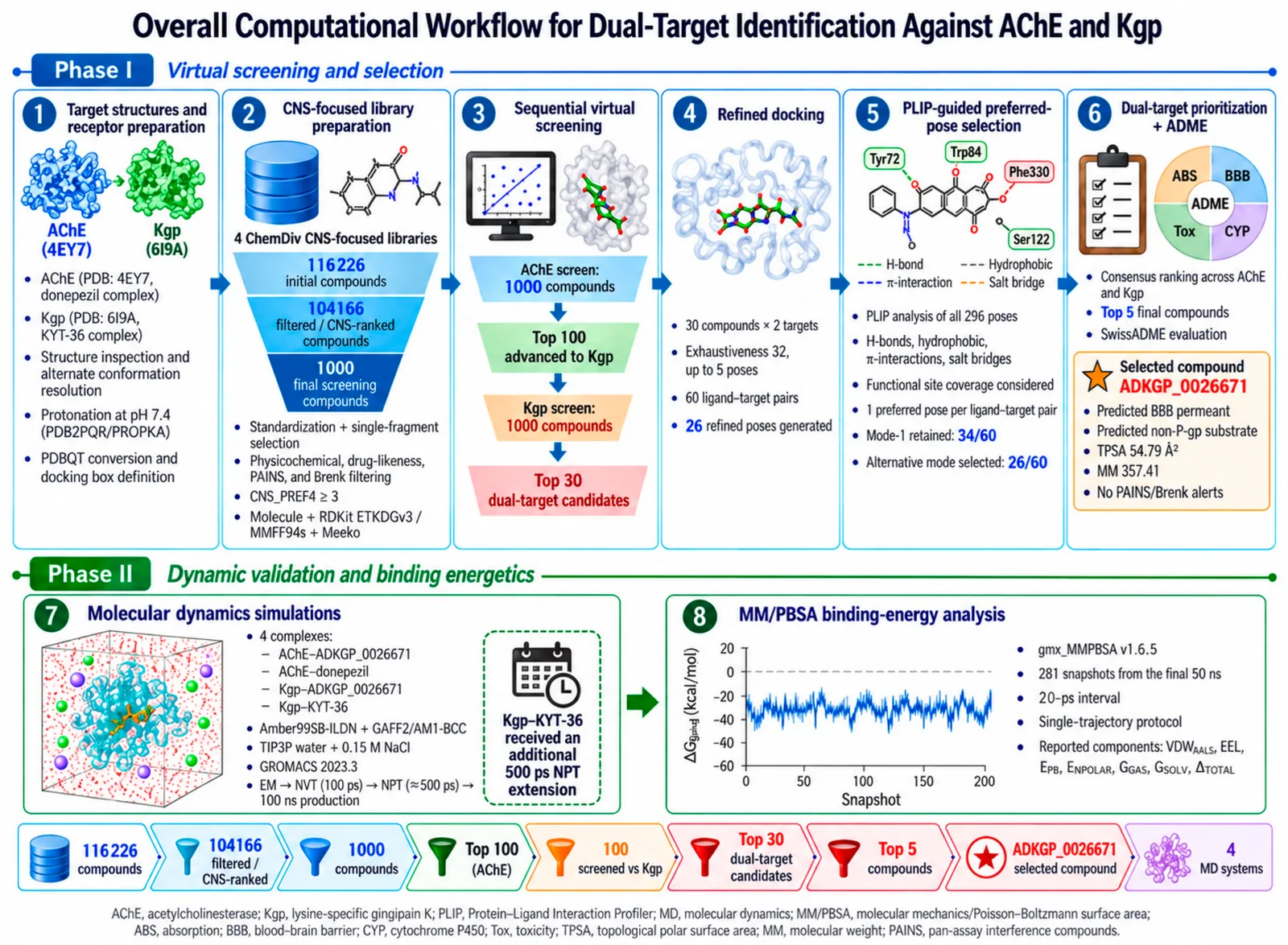
General computational workflow applied to AChE and Kgp for dual-target-based main search via the following steps: receptor-based and neurological-based library preparation, two subsequent virtual screenings, re-docking and PLIP-based preferred pose selection, prioritization based on consensus for dual-target followed by assessment through SwissADME, and finally molecular dynamics study with free energy binding calculations using the MM/PBSA method.

To account for temporal correlation among sequential snapshots, the ΔTOTAL time series was analyzed by autocorrelation analysis. Statistical inefficiency gg was estimated from the initial positive sequence of the autocorrelation function, and the effective sample size was calculated as N_eff_ = N/g. Reported uncertainties represent autocorrelation-corrected standard errors of the trajectory-derived mean, rather than variability among independent simulation replicas. As an additional robustness assessment, block averaging was performed using five approximately 10 ns blocks and ten approximately 5 ns blocks. Because MM/PBSA estimates are method- and system-dependent, absolute ΔTOTAL values were interpreted primarily within each target relative to its corresponding reference inhibitor and were not used to infer quantitative affinity differences between the AChE and Kgp systems. A summary of the overall workflow followed from target and library preparation through screening, PLIP, ADME prioritization, MD, and MM/PBSA is shown in (Figure 7).

## 5. Conclusions

The primary merit of the present study lies in establishing a rational starting point for subsequent validation rather than demonstrating therapeutic efficacy. When interpreting results, it is important to consider certain limitations. Firstly, the study reports dual-target predicted binding potential based on computational analysis alone. In addition, global redocking of KYT-36 into Kgp did not reproduce the crystallographic binding geometry within the conventional RMSD criterion; therefore, Kgp docking-derived poses should be interpreted with corresponding geometric uncertainty, particularly regarding the exact ligand orientation and residue-level interaction patterns. Secondly, the conclusions from molecular dynamics analysis are drawn from a single 100 ns trajectory for each complex without further replicates, limiting inferences about longer-timescale conformational behavior and reproducibility. Finally, though there is emerging evidence linking *P. gingivalis* and gingipains to AD pathology, whether or not they have a causal role in human AD is yet to be established. Therefore, Kgp should only be considered as an investigative target rather than a clinically validated AD target.

Biochemical determination of AChE and Kgp inhibitory activity would constitute the immediate next step, followed by assessments of target selectivity, cytotoxicity, metabolic stability, and experimental permeability across the blood–brain barrier. The dual-target profile in response to exposure to inflammatory or neurotoxic components derived from *P. gingivalis* in cellular models will need verifying alongside whether it translates into any biological meaning, eventually to be verified in appropriate in vivo models to answer the overall question of whether simultaneously modulating the host target of AChE and pathogen virulence factor of Kgp is a viable strategy in tackling the multifactorial biology of AD. In summary, our computational screen has yielded a balanced dual-target hit that bridges symptomatic and microbial pathways in AD. ADKGP_0026671 provides a rational starting point for a novel multi-target ligand strategy. By integrating cholinergic modulation with gingipain inhibition, this work opens a new avenue for AD drug discovery that warrants rigorous experimental follow-up.

## Figures and Tables

**Table 1:** Dual-target drug candidates list. Top five candidates, docking, interaction, and ADME profiles: AChE, acetylcholinesterase; Kgp, lysine-specific gingipain K; MW, molecular weight; TPSA, topological polar surface area; GI, gastrointestinal; BBB, blood–brain barrier; P-gp, P-glycoprotein; PAINS, pan-assay interference compounds; ADME, absorption, distribution, metabolism, and excretion; LogP, logarithm of the octanol/water partition coefficient. MLOGP, Moriguchi logP. ^†^ One Lipinski violation: MLOGP > 4.15.

Compound	Final Rank	AChE Preferred Score (kcal/mol)	Kgp Preferred Score (kcal/mol)	AChE Important Contacts, n	Kgp Catalytic Contact	MW	TPSA	Consensus LogP	GI Absorption	BBB Permeant	P-gp Substrate	Lipinski Violations	PAINS	Brenk
ADKGP_0105683	1	−12.358	−7.159	9	Yes	407.39	85.84	3.74	High	No	No	0	0	0
ADKGP_0001901	2	−12.374	−7.236	6	No	389.47	87.60	3.47	High	No	No	0	0	0
ADKGP_0026671	3	−11.804	−7.345	7	Yes	357.41	54.79	3.88	High	Yes	No	1 ^†^	0	0
ADKGP_0111477	4	−12.539	−7.006	5	Yes	380.35	72.95	3.61	High	Yes	Yes	0	0	0
ADKGP_0079028	5	−12.278	−7.117	7	No	344.36	81.42	2.88	High	No	Yes	0	0	0

**Table 2:** MM/PBSA endpoint binding-energy components for ADKGP_0026671 and the reference inhibitors in AChE and Kgp. VDWAALS, van der Waals energy; EEL, electrostatic energy; EPB, polar Poisson–Boltzmann solvation energy; ENPOLAR, nonpolar solvation energy; GGAS, gas-phase energy; GSOLV, solvation energy; ΔTOTAL, total MM/PBSA endpoint binding-energy estimate; SD, standard deviation; corrected SEM, autocorrelation-corrected standard error of the mean. Energies are reported in kcal/mol. Configurational entropy was not evaluated.

Target	Ligand	VDWAALS	EEL	EPB	ENPOLAR	GGAS	GSOLV	ΔTOTAL	SD	Corrected SEM
AChE	ADKGP_0026671	−44.26	−4.56	34.70	−4.74	−48.82	29.96	−18.86	3.34	0.79
AChE	Donepezil	−50.41	−175.93	200.61	−4.92	−226.33	195.68	−30.65	4.91	0.75
Kgp	ADKGP_0026671	−36.72	−9.79	34.38	−3.54	−46.51	30.84	−15.67	3.56	0.47
Kgp	KYT-36	−42.60	−224.33	228.15	−3.96	−266.93	224.19	−42.74	15.22	2.91

## Data Availability

The data generated and analyzed during this study are included in the article and its Supplementary Materials. Additional data supporting the findings of this study are available from the corresponding author upon request.

## Supplementary Materials

The following supporting information can be downloaded at: https://www.mdpi.com/article/10.3390/ph19101589/s1, Table S1: Dual-target ranking, refined docking scores, interaction-guided pose prioritization, and physicochemical properties of the top 30 candidate compounds; Table S2: Chemical identifiers and structural information for the top 30 dual-target candidate compounds; Table S3: PLIP-derived interaction profiles of the preferred binding poses of the top 30 compounds against AChE and Kgp; Figure S1: Time-resolved contact-occupancy heatmap showing the persistence of ligand–protein interactions during the 100 ns MD simulations; Figure S2: Minimum ligand–pocket distance over time during the 100 ns MD simulations, used to assess maintenance of ligand association with the respective binding pockets.

## Abbreviations

The following abbreviations are used in this manuscript:

AD	Alzheimer’s disease
*P.gingivalis*	*Porphyromonas gingivalis*
Kgp	lysine-specific gingipain
